# Retraction: “Assessing the Dissemination of COVID-19 Articles Across Social Media With Altmetric and PlumX Metrics: Correlational Study”

**DOI:** 10.2196/41544

**Published:** 2022-08-10

**Authors:** Haley N Tornberg, Carine Moezinia, Chapman Wei, Simone A Bernstein, Chaplin Wei, Refka Al-Beyati, Theodore Quan, David J Diemert

**Affiliations:** 1 Department of Medicine Hospital for Special Surgery New York, NY United States; 2 Department of Medicine The George Washington School of Medicine and Health Sciences Washington, DC United States; 3 Department of Psychiatry Washington University School of Medicine and Barnes-Jewish Hospital St. Louis, MO United States; 4 Department of Medicine American University of Antigua Coolidge Antigua and Barbuda; 5 Department of Medicine David Geffen School of Medicine at University of California Los Angeles, CA United States

The authors are retracting “Assessing the Dissemination of COVID-19 Articles Across Social Media With Altmetric and PlumX Metrics: Correlational Study” (J Med Internet Res 2021;23(1):e21408) in alignment with COPE guidelines and based on an honest error that invalidates the results.

Authors’ statement:

We sincerely regret our honest misunderstanding of the Altmetrics variables as it was discussed in our manuscript. As a team that values honest and ethical research, we are grateful to JMIR for bringing this grave error to our attention and appreciate our fellow peers for holding us to the highest standard of research. We strive to uphold integrity in our research and agree with the decision of the editors of JMIR to retract our original manuscript. We look forward to the opportunity to edit our work so our research accurately reflects the intention behind the Altmetrics variables.

